# Hospitalizations of Ukrainian Migrants and Refugees in Poland in the Time of the Russia-Ukraine Conflict

**DOI:** 10.3390/ijerph192013350

**Published:** 2022-10-16

**Authors:** Katarzyna Lewtak, Krzysztof Kanecki, Piotr Tyszko, Paweł Goryński, Irena Kosińska, Anna Poznańska, Michał Rząd, Aneta Nitsch-Osuch

**Affiliations:** 1 Department of Social Medicine and Public Health, Medical University of Warsaw, 3 Oczki Street, 02-007 Warsaw, Poland; 2 Institute of Rural Health in Lublin, 2 Jaczewskiego Street, 20-090 Lublin, Poland; 3 National Institute of Public Health NIH-National Research Institute, 24 Chocimska Street, 00-791 Warsaw, Poland

**Keywords:** refugees’ health, military operations, hospital morbidity, Russian aggression, Ukraine, war refugees

## Abstract

Background: In the face of a sudden influx of several million migrants and war refugees from Ukraine to other European countries, knowledge about the health of Ukrainian citizens becomes increasingly important. The aim of the study is to identify the main health problems of hospitalized Ukrainian citizens residing in Poland in the period from 2014 to June 2022. Methods: This study is based on hospitalization data of Ukrainian patients in Poland taken from the Nationwide General Hospital Morbidity Study. Results: The study group covered 8591 hospitalization records. We observed two hospitalization peaks, one in patients aged 0–5 and the other one in those aged 20–35. After the official outbreak of the war, 2231 Ukrainian citizens were hospitalized in Poland. At this time, the most often reported principal reasons for the hospitalizations of adult women were diseases related to pregnancy, childbirth and the puerperium, whereas in groups of adult men diseases were related to injury, poisoning and certain other consequences of external causes, and in children and adolescents diseases were infectious and parasitic diseases. Conclusions: Our findings may have implications for healthcare policies and service provision to newly arrived migrants and war refugees in target European countries.

## 1. Introduction

Russia’s military operations against Ukraine started in 2014 when Russia annexed Crimea and they continue today, mainly in eastern Ukraine. On February 24, 2022, Russia unreasonably attacked Ukraine. Millions of Ukrainian citizens have left their places of permanent residence and fled to neighboring countries in search of safe living conditions. Ukraine is the second largest European country located in the eastern part of Europe. The population of Ukraine is reported to be about 42 million people.

Upon arrival in foreign countries, wartime immigrants and refugees may require medical assistance. Healthcare needs may be related to the immediate consequences of hostilities, in particular injuries, poisoning, malnutrition, and the lack of proper care in the country of origin. Coronavirus disease (COVID-19), measles, pertussis, tetanus, and poliomyelitis constitute epidemiological risks that can be associated with a rise in the influx of refugees [1]. The COVID-19 pandemic exacerbated Ukraine’s difficulties in the sphere of healthcare, security and governance [2]. It was suggested that the war in Ukraine could be a critical factor in the new outbreak of COVID-19. Such an outbreak could make the already difficult humanitarian situation in Ukraine even worse [3]. In light of the refugee influx, an analysis of dangers to and challenges for the Polish healthcare was made, showing the problems associated with infectious diseases, vaccination coverage in Poland and Ukraine, and vaccinations for refugees, along with the challenges for the Polish healthcare system [4]. One study suggested enhanced HIV prevention and monitoring in the areas where the refugees come to stay [5]. Other European countries have undertaken certain activities related to the influx of refugees from Ukraine. For example, Italy and Germany have offered vaccination to all Ukrainian refugees [6].

In recent years, several million war migrants and refugees have come to Poland from Ukraine. The increase in migration from Ukraine after 2014 was due to the difficult political situation related to Russia’s aggression and its socio-economic consequences. In 2014, according to Statistics Poland, 26.3 thousand work permits for Ukrainian citizens were registered, in 2020—295.3 thousand range (72.6% of all foreigners who received work permits in Poland in 2020) [7,8]. The total refugee influx from Ukraine to Poland was 3,167,805 on 6 May 2022 [9]. According to the United Nations Children’s Fund (UNICEF), during one month of war in Ukraine, as many as 4.3 million children were displaced, 1.8 million of whom crossed the border as refugees [10]. It should be noted that the above-mentioned challenges appeared suddenly and had an unprecedented scale and dynamics. According to the United Nations High Commissioner for Refugees (UNHCR), in the first ten days, since the war started, over 1 million refugees crossed the Polish border, and in the following 12 days, another million refugees came to Poland [11]. The number of Ukrainians staying in Poland before the outbreak of war in 2022 is difficult to establish. However, the scale of migration can be shown using data on work permits in Poland, e.g., 1,760,245 permits were issued in 2020, including 295,272 permanent work permits [8].

Ukrainian citizens have been granted medical care in Poland, where national medical care is publicly funded. Legal solutions aimed at supporting Ukrainian refugees were introduced by the Polish State as early as on day 16 of the Russian hostilities in Ukraine [12]. The Act of 12 March 2022 [12] provides Ukrainian citizens with a range of benefits as for citizens of Member States, i.e., comparable to the Directive 2011/24/EU. This Directive cannot be formally implemented into the legal system in Poland because Ukraine is not a Member State. However, it was implemented de facto. Every citizen of Ukraine legally residing in Poland is guaranteed access to the public healthcare system on the same basis as Polish citizens, excluding health resort treatment and rehabilitation, as well as administration of medicinal products issued to beneficiaries under health policy programmes of the minister competent for healthcare. Ukrainian refugees were entitled to medical care from the date of their legal entry into the territory of Poland. Furthermore, regardless of the possession or lack of documents legalizing the stay in Poland, Ukrainian citizens who are in a state of emergency health are provided free healthcare services in the field of medical rescue operations. These benefits are financed from the state budget. The aim of the study is to identify the main health problems of hospitalized Ukrainian citizens residing in Poland in the period from 2014 to June 2022, including war refugees, on the basis of an analysis of hospital morbidity. The study of the hospital morbidity structure is a valuable source of information on more complex health problems that require a hospital stay. Such data is presented in the statistics of international organizations, including OECD and EUROSTAT [13,14]. An analysis of the health consequences of the war for Ukrainians, based on the hospitalization data of the country that has accepted the largest number of refugees and migrants, may be an important source of information for other countries in taking adjustment measures related to the migration of Ukrainian citizens.

## 2. Materials and Methods

The study is a retrospective study based on data from hospital discharge records containing information that allows for the identification of Ukrainian citizens among hospitalized patients. Data were obtained from the National Institute of Public Health NIH-National Research Institute in Poland and they covered 8591 hospitalization cases reported between January 2014 and June 2022. All hospitals in Poland, except for psychiatric facilities, are required under the law to electronically submit hospitalization data to the Institute. The data are anonymous and they contain information on ICD10-code diagnoses, hospital admission and discharge data, sex, age, date of birth of the patients and country citizenship code. The inclusion criterion was the presence of the Ukraine citizenship code among hospitalized patients. Based on the information obtained from the local bioethics committee, in the case of retrospective and non-invasive research studies, the ethics committee does not issue an opinion. We assumed that diagnoses were made in hospitals on the basis of the most current and widely used diagnostic criteria. Reasons for hospital admissions (principal diagnosis or condition most responsible for the patient’s stay in hospital, determined at discharge from the first hospital ward where the patient was treated) are defined in accordance with the International Statistical Classification of Diseases and Related Health Problems, 10th Revision (ICD-10) and these were subdivided according to their chapter code. The diagnoses from code groups S00-T98 are consequences of external causes, therefore code groups V01-Y98 were not included in the analyses in the study.

To perform the statistical analyses, Statistica (TIBCO Software Inc. (2017) (Palo Alto, CA, USA). Statistica (data analysis software system, version 13) was used. The following statistical measures were computed: means, medians, and ranges for continuous variables, counts and percentages for categorical variables. The statistical analysis was performed using the t-Student test with respect to an assumption of normal distribution in sufficiently large samples in public health research [15] and non-parametric tests (chi square test, Mann–Whitney U-test) if necessary. A two-sided *p* value of less than 0.05 was considered to be statistically significant.

## 3. Results

The study covered 8591 hospitalization records. In the study group, the mean and median age was 29.8 (95% CI: 29.4–30.2, SD: 19.6) and 29 years (IQR: 14–43), respectively. The mean age among males was significantly lower than among females (28.8 vs. 30.7; *p* < 0.0001). Male patients accounted for 48.2% of all patients. Children and adolescents below 18 years of age accounted for 27.3% of all patients. The mean age was significantly higher among patients hospitalized before the official outbreak of the war compared to the group of patients after the outbreak of war (33.6 vs. 18.8, *p* < 0.0001).

**Figure 1:**
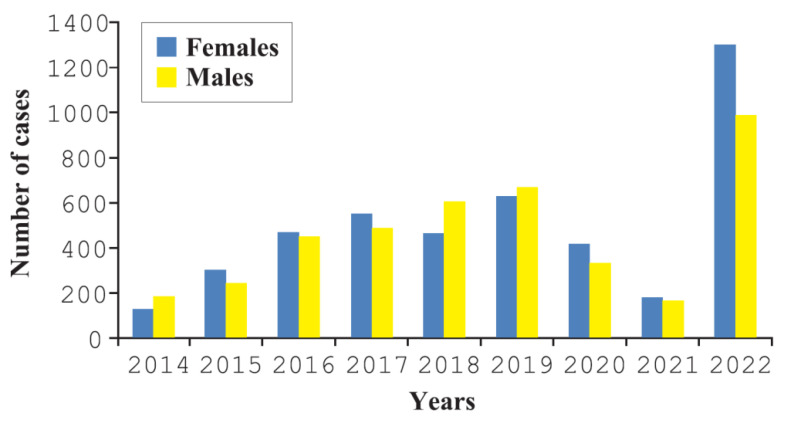
Number of hospitalizations of Ukrainian patients by sex, 2014–2022.

**Figure 2:**
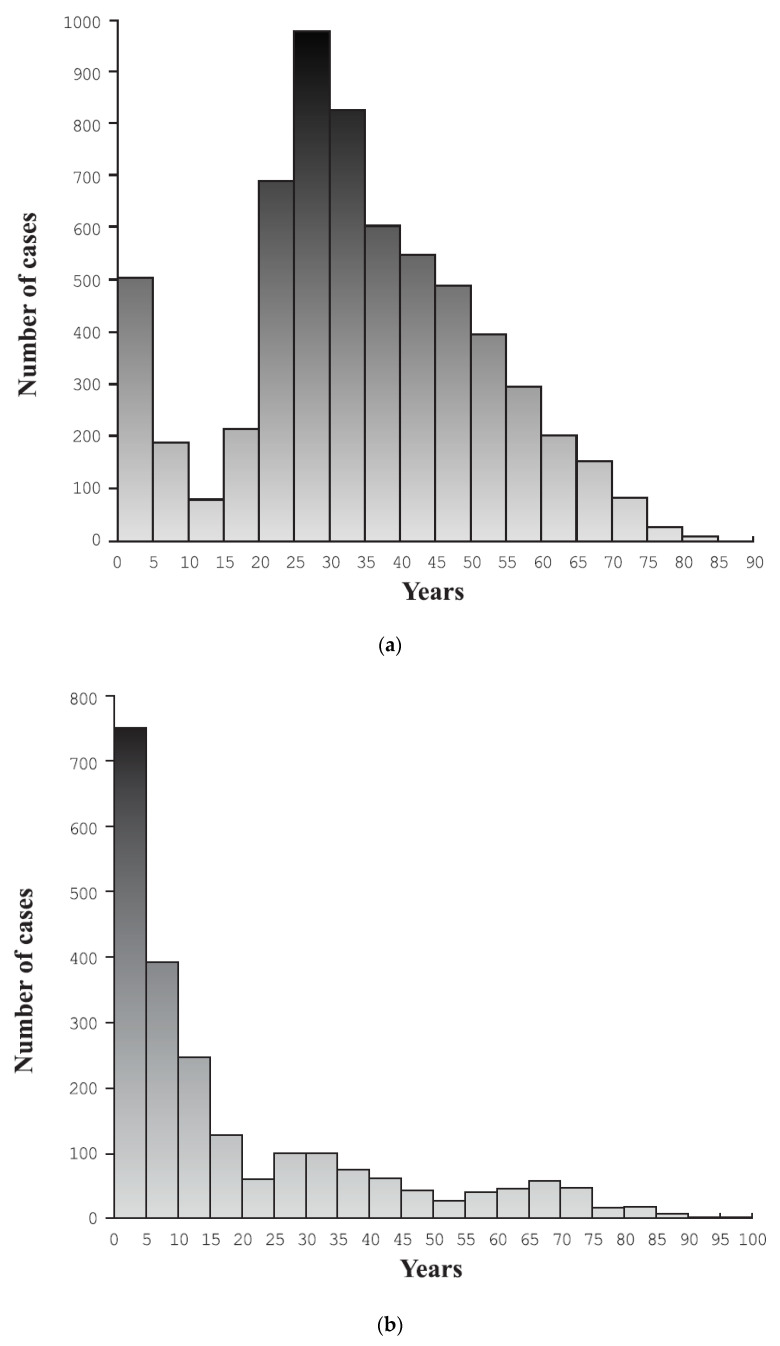
(**a**) Age distribution of Ukrainian patients hospitalized before 24 February 2022. (**b**) Age distribution of Ukrainian patients hospitalized after 24 February 2022.

Figure 1 shows the annual number of hospitalizations of Ukrainians in the studied period in relation to men and women. Age distribution of the study group before and after the outbreak of the war was presented in Figure 2a,b. We observed two hospitalization peaks, one in patients aged 0–5 years and the other one in those aged 20–35 years in the period before the official war outbreak. After outbreak of the war the peak was observed in children under 5 years of age as presented in Figure 2b. 

Table 1 presents the comparative characteristics of the most often reported groups of diseases in the subgroups of patients aged 0–4 years, 5–17 years, adult women and adult men. To better illustrate the differences, a summary of diseases reported before and after the official outbreak of the war is presented. An additional specification concerned the identification of the principal disease as the reason for admission to the hospital, and all diseases found in hospitalized patients. In our study, 2349 hospitalizations (27.3% of all hospitalizations) concerned children and adolescents under 18 years of age. In this subgroup, 1256 hospitalizations concerned children under 5 year of age (14.6% of all hospitalizations).

We observed 74 deaths during hospitalization, which accounts for 0.9% of all hospitalization cases in the study group. The mean and median age were 49.3 years (95% CI: 44.1–55.4, SD: 22.3) and 50 years (IQR: 40–63), respectively. In this group, we observed 45 males and 29 females. The following diseases were most often reported as primary causes of deaths during hospitalization: diseases of the circulatory system—23 cases, neoplasm—16 cases, injury, poisoning and certain other consequences of external causes—10 cases, diseases of the respiratory system—4 cases.

## 4. Discussion

Military operations in Ukraine have caused immigration to Poland and other European countries. Various health consequences have been observed among Ukrainian citizens hospitalized in Poland. Despite Russia’s military actions towards Ukraine since 2014, the first highest number of hospitalizations was recorded in 2019 with subsequent reduction until the official outbreak of the war. The initial intensification of migration may have exacerbated the health needs of Ukrainians, who required hospitalization; however, in the subsequent years there could have been re-emigration to other countries, improvement of the health situation of Ukrainians, adaptation to the Polish healthcare system with the use of outpatient healthcare. Interestingly, later, when the war was officially declared, prompt increase in hospitalizations was observed, as presented in Figure 1. It can be assumed that the changes may have been related to the exacerbation of military operations in Ukraine. 

The official outbreak of the war in Ukraine could significantly change the spectrum of people requiring hospitalization in European countries. Health risks for war refugees are influenced by the epidemiology of noncommunicable and infectious diseases in their countries of origin, the circumstances and conditions of the migration journey and barriers to accessing healthcare after arrival [16]. As presented in Figure 2a,b, the age distribution of hospitalized people in Poland differed significantly, which illustrates how the official outbreak of war can change the age of hospitalized people. Most people fleeing the war and seeking refuge in Poland are likely to be women, children and older adults. Children aged 0–4 required hospitalization at the beginning of this situation. In the period of several years of hostilities before the outbreak of the war, people aged 20–35 often required hospitalization. The presented data can be a clear signal for other countries about what health needs may apply to immigrants and refugees from a few years before and several months after the official outbreak of the war.

The official outbreak of the war caused changes in the morbidity of hospitalized Ukrainians. The presented data show the possible health needs of migrants and refugees during the war. According to the data presented in Table 1, the official outbreak of war may have contributed to the higher incidence of certain infectious and parasitic diseases and diseases of the respiratory system in patients below 18 years of age. It can be assumed that warfare as well as limited access to preventive vaccinations, disease prophylaxis, and the use of effective treatment during war may be the reasons for the occurrence of this group of diseases among Ukrainian patients hospitalized in Poland. One study showed that Ukrainian children were often of ill health and implied that the host countries should always consider the needs of the refugees [17]. According to the information gathered in Table 1, a relatively high percentage of children with respiratory diseases was observed after the war outbreak. It can be assumed that this group of patients may require not only specialist care in a hospital setting, but also an efficient system of medical support and medical care. Poland declared its help and readiness to treat patients from Ukraine within the network of medical support, including Intensive Care Units for both adults and children. Poland offers specialist transport from the country border to hospitals that have offered to admit the most severely ill patients [18]. The presented data concerned health problems of Ukrainian citizens hospitalized in Poland. The results of these analyses may be useful for health sector professionals in other countries that Ukrainian refugees may reach, especially in the context of their health needs. 

Among adult women, pregnancy, childbirth and the puerperium were the most common major causes of hospitalization both before and after the official outbreak of the war. Immigrant women from the conflict zones were at a higher risk of neonatal mortality and morbidity [19]. A certain link was observed between the infections of women’s reproductive tracts related to healthcare and a miscarriage history [20]. The Russian invasion of Ukraine has had an unprecedented impact on the mental health of people who have taken refuge in Poland and other European countries. It has been reported that children and families who live in or run away from war zones are more likely to suffer from mental health disorders than the general population [21]. 

It can be assumed that the most common principal reasons for hospitalization among adult male immigrants and war refugees were health problems related to injury, poisoning and certain other consequences of external causes (ICD-10 code: S00-T88). Interestingly, after the official outbreak of the war, this group of diseases continued to dominate, however to a lesser extent, which may suggest that the intensification of hostilities could have increased the number of wounded men in the country covered by the war, but also limited the possibility of migration for hospital treatment. 

In a recent study on Ukrainian hospitals, it was reported that in some cases hospitals were overloaded with injured people, and mild to moderately injured patients were transferred to nearby hospitals. If a high number of persons are injured in heavy fighting or explosions, the rules of triage are followed and patients whose injuries are mild or moderate are routed to the second line hospitals [22]. It may be highly probable that a part of these patients were hospitalized in Poland.

Being one of the main reasons for the hospitalization of adult Ukrainians, injuries and wounds could also be related to the presence of infections. It may be difficult to successfully provide medical help to Ukrainian patients with war injuries due to possible antibiotic resistance, and changes in the epidemiology of wound infection that occur over time [23,24,25,26]. Thus, some patients could be referred to other countries, including Poland, in order to obtain extended treatment options, not available in Ukraine.

Among principal cases of hospitalizations in adult patients after the war outbreak, neoplastic diseases were reported. In many ways, war and cancer are linked. War delays cancer diagnoses, preventing treatment, and increasing risk. War exposes vulnerable patients with cancer to infections and threatening conditions [27]. It can be supposed that a part of Ukrainian citizens did not have the possibility to continue the oncological treatment at home, or that due to the current military operations in Ukraine, it was not possible to undertake effective diagnostics and treatment. Neoplasms, as one of the principal causes of hospitalization, were also reported in children 5–17 years of age before the official outbreak of the war, as reported in Table 1. Oncological treatment in Ukraine has been reported to be hindered, and it is necessary to globally support Ukrainian patients [28,29]. Some studies indicate that mortality related to cancer will increase [30]. Breast, corpus uteri and colorectal cancer were the most frequently reported types of cancer in Ukrainian female patients in 2020 [31]. Since many patients are not given cancer-specific treatment, the incidence of colorectal cancer in Ukraine is expected to rise [32]. It is worth emphasizing the role of Ukrainian and European oncologists who struggled to coordinate patient care and allocation across borders. While health centers in Poland were reported overcrowded, centers in other countries were not fully engaged [33]. As soon as a few days after the outbreak of the war, the Polish Society of Gynecologic Oncology launched the coordination of the treatment of Ukrainian patients suffering from the gynecological cancers [34]. Polish National Health Fund, National Health Fund cooperating with 20 top Polish comprehensive cancer centers, established official hotline for oncological patients from Ukraine. Oncology patients from Ukraine could be directed to the correct hospital treating the given type of cancer [35]. The global oncologic community (e.g., societies, universities, nongovernmental organizations, and institutions responsible for international public health), must work together to help cancer patients and provide the care they deserve and urgently require.

Other differences in principal causes of hospitalizations were also reported, as presented in Table 1, which suggests that the official start of hostilities in Ukraine may also affect other health problems. To better illustrate the health situation of hospitalized Ukrainians, data on the most common groups of diseases are presented in relation to all reported diseases. The most frequently reported principal diagnoses for hospitalization were similar to the most frequent diagnoses taken from all reported diagnosis. Diseases of the genitourinary system were among the most frequently reported hospitalization causes only in the group of adult women in the period before the war outbreak. It is worth to be highlighted that it this situation countries such as Poland, Italy, Germany and others offered medical help to hemodialysis patients [36].

Among those hospitalized after the official outbreak of the war, more children and adolescents and fewer adults were observed than before the outbreak, as presented in Table 1. The healthcare services of the target countries should take into consideration the possibility of such changes and should adapt the healthcare in good time to the hospitalization of children and adolescents, the most vulnerable victims of any war.

The percentage of people who died during hospitalization was small. The majority were men, and more than half of the underlying causes of death were related to cardiovascular diseases and neoplasms. These data suggest which patient groups are at risk of unfavorable prognosis during hospitalization and can form the basis for targeted healthcare organization in the target country.

The study presented in this paper has several limitations. The database, which it was based on, and allowed the authors to make this report, was not assessed with regard to the reporting and coding practices for hospital diagnoses. Considering the type and scope of available data, a detailed analysis of the hospitalization reasons was not assessed in this study. The database includes only records that have been reported to the national hospital morbidity register, and concern people who have been assigned the Ukrainian citizenship code. If the patient was not reported with the code of a Ukrainian citizen, they were not included in this study. This may have limited the actual number of Ukrainian citizens hospitalized in Poland. The war in Ukraine and its significant burden on the Polish healthcare system due to the influx of refugees and immigrants from this country could have affected the efficiency of reporting hospitalization cases, especially in the last year. The study group includes Ukrainian citizens who have arrived in Poland as war refugees, and Ukrainian citizens who came to Poland for other reasons, both before the start of the war and after the outbreak of the Russian-Ukrainian war. It was not possible to precisely differentiate between these groups of Ukrainian citizens in the study. However, the significant number of several million immigrants and war refugees from Ukraine who have come to Poland in recent years may indicate that the results of the study mainly describe immigrants and war refugees. In the database, there was no information on the date of birth and age in 17 hospitalization cases, and in one case patient sex was not reported as male or female. A part of the multi-million group of Ukrainian citizens may not have had health insurance, and some hospitalization cases could concern mainly emergencies.

Recently, inequalities in access to healthcare according to the local policy model among newly arrived refugees were reported [37]. Results from our study may be the basis for incorporating the needs of refugees and migrants in national and local health policies, as well as supporting access to healthcare for war refugees and migrants. 

The data presented in this paper concern the spectrum of hospital morbidity of Ukrainian citizens hospitalized in Poland during the period of military operations in Ukraine. The spectrum of disease prevalence varies from one patient group to another. European countries may experience similar problems as Poland in light of the incoming war refugees and immigrants from Ukraine. The data presented could be useful for other European countries when developing effective strategies to help Ukrainian citizens and mitigate public health issues.

## 5. Conclusions

The so-far unprecedented large influx of refugees and migrants from Ukraine, which has been experienced by European countries for several years, has put the issue of migration at the forefront of the political agenda in many European countries. The influx of this group of people may overload healthcare systems in European countries, especially in terms of health needs related to hospitalization. There is no recent study on the burden of hospitalizations in a country that has welcomed a group of several million immigrants and refugees in recent years.

The results of the conducted analyzes provide important knowledge about changes in the intensity and structure of hospital morbidity of Ukrainian migrants and war refugees. 

A short-term solution was presented for the early management of specific diseases in the groups of migrants and refugees. Strategies should focus primarily on the prevention and control of infectious diseases (testing, vaccination and implementation of protective measures), screening and treatment of noncommunicable diseases, as well as supporting their access to healthcare. 

A long-term solution was presented for a better understanding of the health of migrants internationally, which will support the development of health policies and improve the continuity of healthcare in target countries. There is an urgent need to protect war refugees and other migrants, meet their health needs, and build responsive health systems in host communities.

Poland’s experience in this area is unique and may be useful for other European countries. This research opens the door for future research in this area.

## Figures and Tables

**Table 1:** Number and percentage of main hospitalization diagnoses in the study group.

	Before Official War Outbreak (January 2014–23 February 2022)				After the Official War Outbreak (24 February 2022–30 June 2022)			
Age	0–4 Years	5–17 Years	Adult Women	Adult Men	0–4 Years	5–17 Years	Adult Women	Adult Men
Number of cases	507	348	2859	2623	749	745	578	158
Principal diagnosis reported (ICD-10 *)	Z00–Z99 245 (48%) P00–P96 154 (30%) Q00–Q99 29 (6%) J00–J99 19 (4%)	D50–D89 70 (20%) S00–T98 50 (14%) E00–E90 34 (10%) C00–D49 28 (8%)	O00–O99 1175 (41%) S00–T98 357 (12%) C00–D49 267 (9%) N00–N99 252 (9%)	S00–T98 1224 (47%) I00–I99 266 (10%) K00–K95 190 (7%) R00–R99 184 (7%)	A00–B99 224 (30%) J00–J99 113 (15%) R00–R99 99 (13%) Z00–Z99 55 (7%)	A00–B99 151 (20%) S00–T98 106 (14%) R00–R99 96 (13%) J00–J99 63 (8%)	O00–O99 228 (39%) C00–D49 81 (14%) K00–K93 39 (7%) I00–I99 36 (6%)	S00–T98 27 (17%) I00–I99 25 (16%) C00–D49 24 (15%) H00–H95 14 (9%)
All reported diagnosis (ICD-10 *)	Z00–Z99 271 (53%) P00–P96 167 (33%) Q00–Q99 37 (7%) J00–J99 21 (4%)	D50–D89 123 (35%) C00–D49 70 (20%) Z00–Z99 70 (20%) S00–T98 53 (15%)	O00–O99 1186 (42%) C00–D49 425 (15%) S00–T98 365 (13%) N00–N99 301 (11%)	S00–T98 1258 (48%) I00–I99 334 (13%) K00–K93 244 (9%) R00–R99 224 (9%)	A00–B99 319 (43%) J00–J99 182 (24%) R00–R99 124 (17%) Z00–Z99 85 (11%)	A00–B99 212 (28%) R00–R99 129 (17%) S00–T98 116 (16%) E00–E90 100 (13%)	O00–O99 228 (39%) C00–D49 108 (19%) Z00–Z99 69 (12%) I00–I99 62 (11%)	I00–I99 41 (26%) S00–T98 34 (22%) C00–D49 29 (18%) Z00–Z99 21 (13%)

* Abbreviations of groups of ICD-10 codes reported in the abovementioned table: A00–B99: Certain infectious and parasitic diseases; C00–D49: Neoplasms; D50–D89: Diseases of the blood and blood-forming organs and certain disorders involving the immune mechanism; E00–E90: Endocrine, nutritional and metabolic diseases; H00–H95: Diseases of the eye and adnexa + Diseases of the ear and mastoid process; I00–I99: Diseases of the circulatory system; J00–J99: Diseases of the respiratory system; K00–K95: Diseases of the digestive system; N00–N99: Diseases of the genitourinary system; O00–O99: Pregnancy, childbirth and the puerperium; P00–P96: Certain conditions originating in the perinatal period; Q00–Q99: Congenital malformations, deformations and chromosomal abnormalities; R00–R99: Symptoms, signs and abnormal clinical and laboratory findings, not elsewhere classified; S00–T98: Injury, poisoning and certain other consequences of external causes; and Z00–Z99: Factors influencing health status and contact with health services.

## Data Availability

All technical and substantive information regarding the data used in this work is available on the website of the National Institute of Public Health NIH-National Research Institute. Website: http://www.statystyka.medstat.waw.pl/Informacyjna.html (accessed on 1 August 2022). The data is collected as part of official public statistics.
